# Screening of different interactions in oxo-manganese porphyrin dimers containing axial N-donor ligands: a theoretical study†

**DOI:** 10.1039/c8ra00540k

**Published:** 2018-03-08

**Authors:** Hossein Kavousi, Abdolreza Rezaeifard, Heidar Raeisi, Maasoumeh Jafarpour

**Affiliations:** Department of Chemistry, Faculty of Science, University of Birjand Birjand 97179-414 Iran rrezaeifard@birjand.ac.ir +98 561 32202515 +98 561 32202516

## Abstract

A theoretical analysis for describing the dimeric assemblies of high-valent manganese(v)-oxo *meso*-tetraphenylporphyrin (TPP) ([(TPP)Mn^V^O]_2_^2+^) and *meso*-tetrakis(pentafluorophenyl)porphyrin (TPFPP) ([(TPFPP)Mn^V^O]_2_^2+^) in the presence of axial N-donor ligands is presented. Our theoretical results revealed two types interactions in dimers: a sandwich-like interaction between phenyl rings of porphyrin molecules, and a non-bonded T-shape interaction between nitrogen donors attached to Mn centers. The curvature in the geometry of porphyrin in the [(TPP)Mn^V^O]_2_^2+^/N-donor system is significantly smaller than that of [(TPFPP)Mn^V^O]_2_^2+^/N-donor system. Moreover, the Mn–N_(ax)_ distances in [(TPFPP)Mn^V^O]_2_^2+^/N-donor system are shorter than those of [(TPP)Mn^V^O]_2_^2+^/N-donor system. Also, the donor–acceptor interaction between the imidazoles and the Mn centers are stronger than those of the other ligands in both porphyrins. These results are supported by atoms in molecules (AIM) and natural bond orbital (NBO) analysis.

## Abstract 2

A DFT analysis for describing the dimeric assemblies of high-valent manganese(v)-oxo *meso*-tetraphenylporphyrin ([(TPP)Mn^V^O]_2_^2+^) and *meso*-tetrakis(pentafluorophenyl)porphyrin ([(TPFPP)Mn^V^O]_2_^2+^) in the presence of axial N-donor ligands is presented.

## Graphical abstract

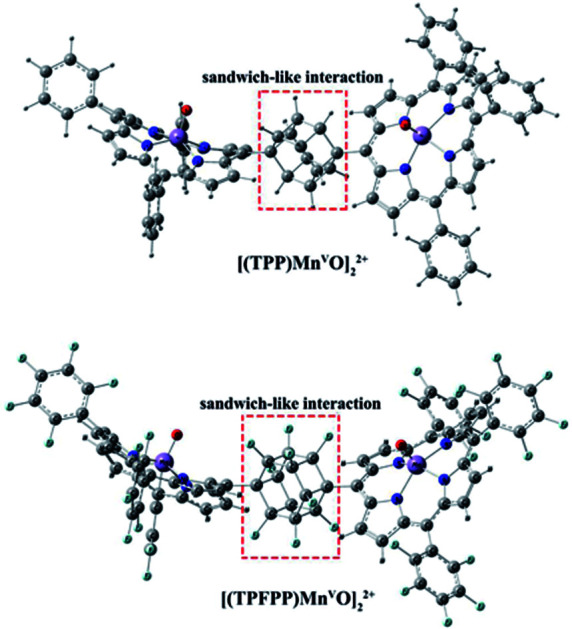

## 1. Introduction

Multiporphyrin arrays have a wide range of potential applications in areas such as light harvesting, nonlinear optics (NLO), organic light-emitting diodes (OLEDs), and photodynamic therapy (PDT). Hence, these systems have been investigated widely.^1–3^ A number of porphyrin dimers have been reported as promising viscosity-sensitive molecular rotors, efficient electrocatalytic CO_2_ to CO conversion, dimer-sensitized solar cells, as ionophores in chemical sensing of anions and cations.^4–6^ π-Conjugated molecules with a small highest occupied molecular orbital–lowest unoccupied molecular orbital (HOMO–LUMO) gap have also been studied with regard to their potential use as molecular wires in molecular-scale electronics and nanotechnological devices.^7^ Porphyrin nanoparticles are favorable apparatuses of advanced materials because of the rich photochemistry, stability, and confirmed catalytic activity.^8^ In analogy to inorganic and other organic nanoparticles, it is predictable that nanoparticles of porphyrins will have unique photonic properties not accessible by larger-scaled materials having the macrocycle, or by the molecules themselves.^9,10^

The promising results obtained in our recent work on the synthesis and catalytic oxidation activity of Mn–porphyrin nanoparticles^11^ induced us to investigate a computational density functional theory (DFT) study to describe the effect of diverse agents on the structure and activity of manganese(v)-oxo *meso*-tetraphenylporphyrin.^12–14^ Herein, new porphyrinic dimers comprising two distinct oxo-Mn–porphyrin complexes containing nitrogen donors is designed which are linked to each other through phenyl/phenyl as well as axial ligands interactions. The main purpose of this research is a DFT study to investigate the interactions between high-valent manganese(v)-oxo *meso*-tetraphenyl porphyrin dimer ([(TPP)Mn^V^O]_2_^2+^) and *meso*-tetrakis(pentaflourophenyl) porphyrin dimer ([(TPFPP)Mn^V^O]_2_^2+^). The interactions between nitrogen donors coordinated to Mn centers of [(TPP)Mn^V^O]_2_^2+^ and [(TPFPP)Mn^V^O]_2_^2+^ dimer structures are also investigated.

## 2. Computational methodology

The Gaussian 09W program suite^15^ was used for all quantum chemistry computations. The electronic geometries of all systems were fully optimized in their ground states in the gas phase, using the B3LYP/6-31G (d) level of theory^16–18^ and the relativistic effective core potentials of Mn atom are considered using the LANL2DZ (Los Alamos National Laboratory 2 double ζ) as extra basis.^19^ Approximate distances between the phenyl rings in the complexes investigated have been assessed by introducing ‘ghost atoms’ in geometric centers of the rings and measurement of the distances between them. The frequency calculations were performed at the same level to verify whether stationary points from geometry optimization calculations were local minima or saddle points. The binding energies were corrected for the basis set superposition error (BSSE) by the Boys–Bernardi counterpoise technique.^20^ The procedure for obtaining the adsorption energy is as follows:1
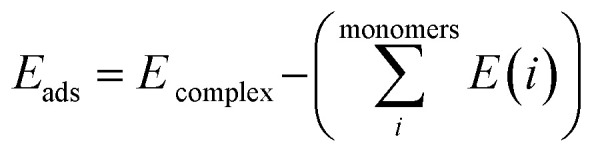
where 
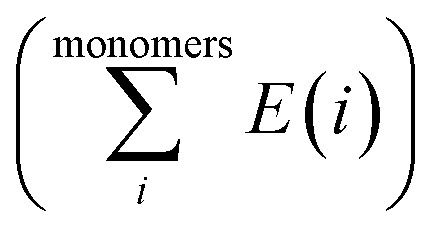
 is the sum of electronic energies of the fully optimized geometries of isolated monomers, while *E*_complex_ denotes the electronic energy of a system studied. It should be mentioned that the adsorption energy encompasses both interaction (*E*_int_) and deformation (*E*_def_) energy contributions, which are both occurred during the adsorption process. Hence, the following equations are applied to calculate these contributions:

2 *E*_ads_ = *E*_def_ + *E*_int_

3 *E*_int_ = *E*_complex_ − *E*_monomers in complex_

The natural bond orbital (NBO) analysis^21^ is also conducted on optimized geometries with the NBO 3.1 included in Gaussian 09. In order to understand the nature of bonding between the constituent atoms, the topological properties of the complexes have been calculated using the atoms-in-molecules (AIM) approach^22^ with MULTIWFN software.^23^

## 3. Results and discussion

### 3.1. Optimized geometries and energetic

**Fig. 1:**
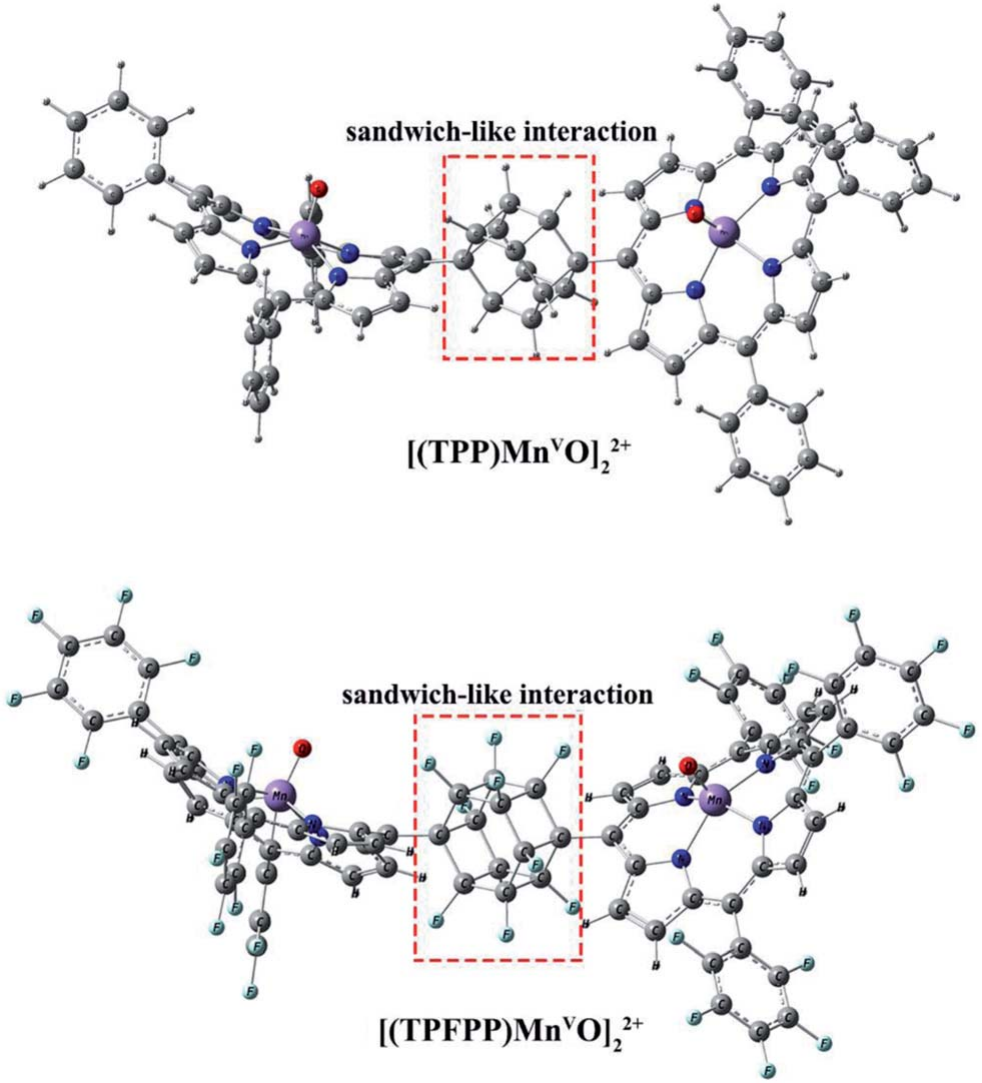
Gas phase DFT optimized structure of the [(TPP)MnO]_2_^2+^ and [(TPFPP)MnO]_2_^2+^.

**Fig. 2:**
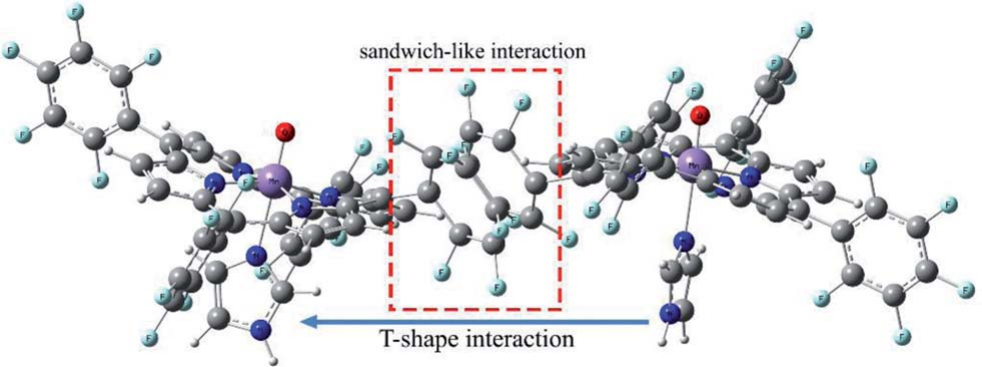
Gas phase DFT optimized structure of the [(TPFPP)(ImH)MnO]_2_^2+^.

Geometric analysis of [(TPP)Mn^V^O]_2_^2+^ and [(TPFPP)Mn^V^O]_2_^2+^ structures shows the existence of sandwich-like interaction between the phenyl rings at the *meso*-positions (Fig. 1). We found that in the energy minima geometries the distance between sandwiched aromatic rings is about 1.72 Å which is about a single C–C bond. The torsion angle between phenyl groups and porphyrin plan is about 93°. The structures were optimized in association with different N-donor ligands (imidazole, pyridine and piperidin). The sandwich-like interaction between phenyl rings of porphyrin molecules as well as a non-bonded T-shape interaction between nitrogen donors attached to Mn centers of porphyrins were identified as the most energetically stable geometries (an example show in Fig. 2). The calculated bond distances, bond angles, torsion angles, adsorption energy (*E*_ads_), interaction energy (*E*_int_) and deformation energy (*E*_def_) at the B3LYP level for[(TPP)Mn^V^O]_2_^2+^, [(TPFPP)Mn^V^O]_2_^2+^and their derivatives are listed in Table 1.

**Table 1:** The selected geometric parameters (*r* and *θ* is in Å and °), torsion angle between the phenyl groups and porphyrin plan (*φ*), adsorption energy (*E*_ads_), interaction (*E*_int_) and deformation (*E*_def_) energy in kcal mol^−1^ for [(TPP)(N-donors) MnO]_2_^2+^ and [(TPFPP)(N-donors) MnO]_2_^2+^dimers calculated in the gas phase^a^

Porphyrin	N-donor	Mn–N_(ax)_	Mn–O	*φ*	*E*_ads_	*E*_inter_	*E*_def_
TPP	ImH	2.370	1.520	95.1	−662.7	−902.1	239.4
	Py	2.538	1.518	94.4	−681.2	−915.4	234.2
	Piperidine	2.547	1.521	93.7	−768.7	−1004.7	236.0
	None	—	1.523	93.5	−563.9	−798.5	234.6
TPFPP	ImH	2.316	1.517	93.8	−710.8	−2624.1	1913.3
	Py	2.472	1.516	93.5	−3101.7	−11347.7	8246.1
	Piperidine	2.508	1.518	93.2	−756.0	−2607.3	1851.3
	None	—	1.516	93.9	−313.7	−1935.5	1621.8

a TPP, *meso*-tetra-phenylporphyrin; TPFPP, *meso*-tetra-pentafluorophenylporphyrin; ImH, imidazole; Py, pyridine.

The Mn–N_(ax)_ distances of the [(TPP)Mn^V^O]_2_^2+^ and [(TPFPP)Mn^V^O]_2_^2+^ in the presence of different nitrogen donors are found to vary in the order ImH < Py < piperidine in the gas phase. Also, the Mn–N_(ax)_ distances of nitrogen donors in association with [(TPFP)Mn^V^O]_2_^2+^ are shorter than those in association with [(TPP)Mn^V^O]_2_^2+^. Thus, the manganese(v)-oxo species in the [(TPFP)Mn^V^O]_2_^2+^/N-donors systems are expected to be more active than the [(TPP)Mn^V^O]_2_^2+^/N-donors systems as evidenced by its oxidation activity (cyclooctene epoxidation yield are 27 and 85% for Mn(TPP)OAc and Mn(TPFPP)OAc nanoparticles, respectively with ImH as an axial ligand^11^). The shortest Mn–N_(ax)_ distance was observed for [(TPFPP)Mn^V^O]_2_^2+^ complex in the presence of ImH, while, [(TPP)Mn^V^O]_2_^2+^ complex has the longest Mn–N_(ax)_ distance in association with piperidine. According to results given in Table 1, adsorption energy (*E*_ads_) values are negative, which means that the adsorption process is favorable. The most negative interaction energy belongs to the [(TPFPP)Mn^V^O]_2_^2+^ in combination with pyridine with −11 347.7 kcal mol^−1^. It means that T-shape interaction between pyridines coordinated to Mn centers of [(TPFPP)Mn^V^O]_2_^2+^is more favorable than the other derivatives. The adsorption energy encompasses interaction (*E*_int_) and deformation (*E*_def_) energy contributions, both of which occur during the adsorption process. The deformation and interaction energies of these systems were calculated (Table 1). Inspection of the results in Table 1 clearly shows that the deformation energy of porphyrin ring in the [(TPP)Mn^V^O]_2_^2+^/N-donor system is less than the [(TPFPP)Mn^V^O]_2_^2+^/N-donor system, which means that the curvature in the geometry of porphyrin in the [(TPP)Mn^V^O]_2_^2+^/N-donor system is significantly smaller than the [(TPFPP)Mn^V^O]_2_^2+^/N-donor system.

### 3.2. Frontier molecular orbital analysis

The energy difference between the highest occupied (HOMO) and the lowest unoccupied (LUMO) molecular orbitals, Δ*E*_(HOMO–LUMO)_, chemical hardness and chemical potential of [(TPP)Mn^V^O]_2_^2+^, [(TPFPP)Mn^V^O]_2_^2+^ and their derivatives are given in Table S1 (in ESI†). The molecular orbital spatial distributions of HOMO and LUMO for [(TPP)(ImH)Mn^V^O]_2_^2+^, [(TPP)(Py)Mn^V^O]_2_^2+^, [(TPFPP)(ImH)Mn^V^O]_2_^2+^ and [(TPFPP)(Py)Mn^V^O]_2_^2+^ are shown in Fig. S1 (ESI†). According to Fig. S1,† the HOMO and LUMO of [(TPP)Mn^V^O]_2_^2+^ species concentrate in the center of both porphyrin rings, whereas the HOMO and LUMO of [(TPFPP)Mn^V^O]_2_^2+^ species concentrate only in the center of one porphyrin ring. According to Koopmans's theorem, the ionization potential is simply the orbital energy of the HOMO, with change in sign. For spin-paired molecules, the electron affinity is the negative of the orbital energy of the LUMO; therefore, for a molecule, the chemical potential (*μ*) and global hardness (*η*) can be calculated as follows:^24–26^4
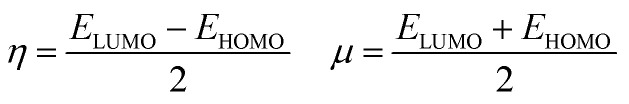


Thus a hard molecule has a large energy gap, and a soft one provides a small gap. According to the data presented in Table S1,† the axial bases generate a softer oxo species. Furthermore, the [(TPP)Mn^V^O]_2_^2+^ species are softer than the [(TPFPP)Mn^V^O]_2_^2+^ species.

### 3.3. Atoms-in-molecules (AIM) topological analysis

**Fig. 3:**
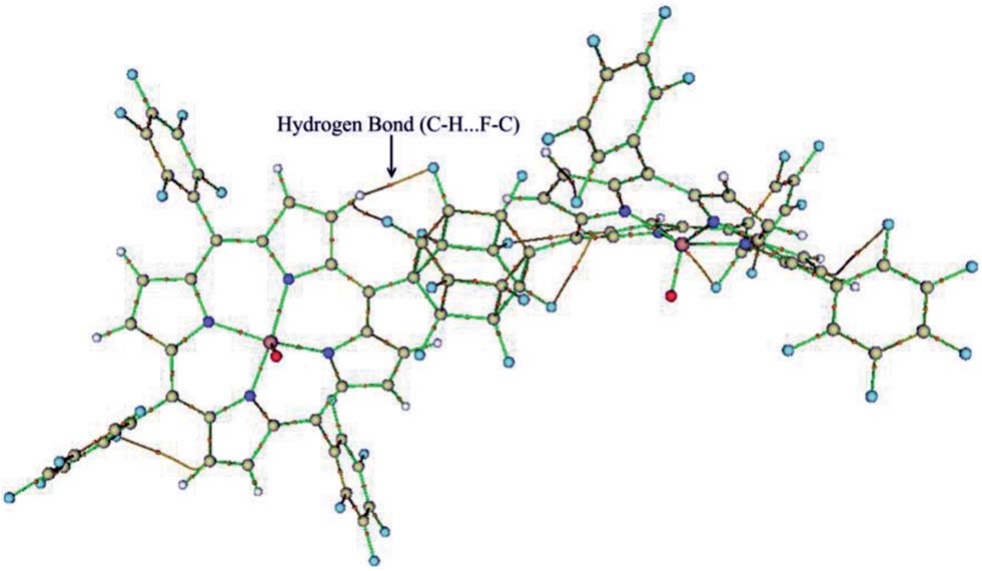
Molecular graph of [(TPFP)Mn^V^O]_2_^2+^ which display C–H⋯F–C hydrogen bonds.

The quantum theory of ‘‘atoms in molecules’’ (QTAIM)^22^ was applied in this study to find critical points (CP) and further to analyze them in terms of electron densities, Laplacians and the total electron energy density *H*_C_. The QTAIM topological results are summarized in Table S2 (ESI†). Inspection of the results in Table S2,† shows the relatively high *ρ* (ranging from 0.3220–0.3276) and ∇_*ρ*_BCP__^2^ (ranging from 1.1319–1.1902) for Mn–O bonds with *H*_C_ < 0, demonstrating covalent bonds with medium strength or even more. But, the Mn–N_(ax)_ bonds have low *ρ* (ranging from 0.0270–0.0453) and positive ∇_*ρ*_BCP__^2^ (ranging from 0.0768–0.1514), which reveal an electrostatic nature in the bonds. It is in line with the classification of bonds given by Rozas *et al.*^27^ The electron density of Mn–N_(ax)_ for [(TPFP)Mn^V^O]_2_^2+^/N-donors systems are more than corresponding values for [(TPP)Mn^V^O]_2_^2+^/N-donors systems. These results are associated with the shorter distances of Mn–N_(ax)_ for [(TPFP)Mn^V^O]_2_^2+^/N-donors systems, which are in complete agreement with geometrical results. Moreover, some evidences for interaction of fluorine groups with the pyrolic hydrogens of [(TPFP)Mn^V^O]_2_^2+^ are also observed (Fig. 3). These interactions may be responsible for more curved structure of [(TPFP)Mn^V^O]_2_^2+^ species.

### 3.4. Natural bond orbital (NBO) analyses

To find more exact information about the nature of these systems, further study is devoted to the NBO analysis.^21^ The results presented in Table S3 (ESI†) shows second-order perturbation stabilization energies, *E*^(2)^, corresponding to charge transfer between nitrogen lone pair of axial ligand and 
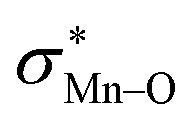
 orbital 
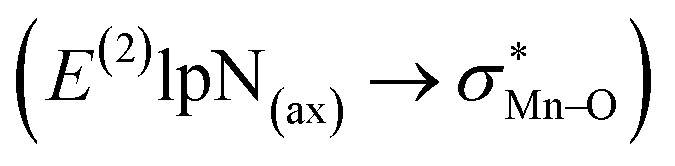
. The maximum and minimum values of the second-order perturbation stabilization energies 
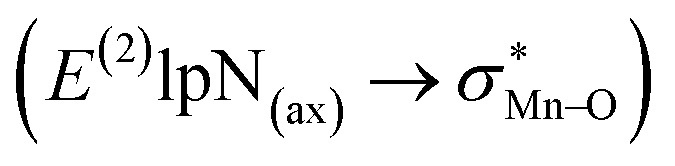
 correspond to [(TPFPP)(ImH)Mn^V^O]_2_^2+^ and [(TPP)(Py)Mn^V^O]_2_^2+^ complexes, respectively. The *E*^(2)^ parameter can be taken as an index to estimate the relative strength of the electron donation of the axial ligand to the Mn–O bond. The values of 
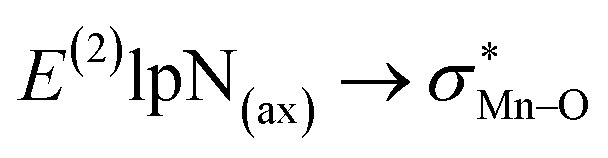
 in [(TPFPP)(N-donors) Mn^V^O]_2_^2+^ systems are more than pertinent values in [(TPP)(N-donors)Mn^V^O]_2_^2+^ systems. These results once again confirm that the donor–acceptor interaction between the N-donors and the Mn centers of [(TPFPP)Mn^V^O]_2_^2+^ are stronger than [(TPP)Mn^V^O]_2_^2+^ species. Also, the donor–acceptor interaction between the imidazole and the Mn centers is stronger than those of the other ligands in association with the both porphyrins. Furthermore, our results show that non-bonded T-shape interaction between N-donors has a significant negative effect on their donor ability. For example, pyridines with a strong T-shape interaction exhibited weaker donor ability than that of piperidine in association with both [(TPFPP)Mn^V^O]_2_^2+^ and [(TPP)Mn^V^O]_2_^2+^ species. (
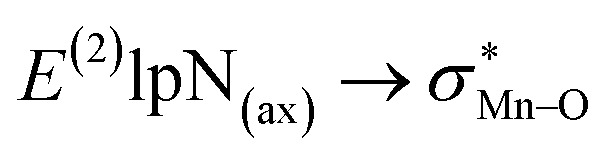
 for pyridines is less than other N-donors).

## 4. Conclusion

In this study, a sandwich-like interaction between phenyl rings of porphyrin ligands and a T-shape configuration interaction between nitrogen donors attached to Mn centers were described. The geometries, electronic structures, vibrational frequencies and physical properties such as dipole moment, chemical potential, and chemical hardness of [(TPFPP)Mn^V^O]_2_^2+^ and [(TPP)Mn^V^O]_2_^2+^ in combination with three classes of nitrogen donors were investigated in the gas phase using DFT calculations. Our results showed that when the N-donor ligands were coordinated to the Mn centers of porphyrin complexes, the less deformation energy was observed for [(TPP)Mn^V^O]^2+^ dimers than that of [(TPFPP)Mn^V^O]^2+^ dimers. The maximum interaction between nitrogen donors belongs to pyridine. [(TPFPP)(ImH)Mn^V^O]_2_^2+^ exhibited the maximum second-order perturbation stabilization energies 
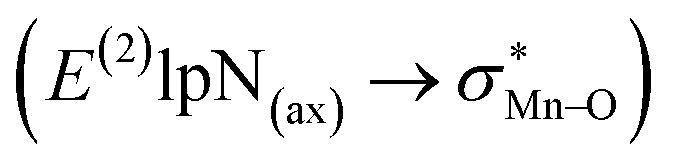
. Furthermore, the geometrical results are nicely confirmed by the AIM data.

## Conflicts of interest

There are no conflicts to declare.

## Supplementary Material

RA-008-C8RA00540K-s001
