# Supplementary material for: Screening of different interactions in oxo-manganese porphyrin dimers containing axial N-donor ligands: a theoretical study
Source: RSC Adv. 2018 Mar 8;8(18):9770–4. doi: 10.1039/c8ra00540k (PMC9078716; doi:10.1039/c8ra00540k)
Supplement: RA-008-C8RA00540K-s001 [file RA-008-C8RA00540K-s001.pdf]

Electronic Supplementary Material (ESI) for RSC Advances.
This journal is © The Royal Society of Chemistry 2018

Supporting Information

Screening of different interactions in oxo-manganese porphyrin dimers containing axial N-donor ligands: A theoretical study

Hossein Kavousi, Abdolreza Rezaeifard,* Heidar Raeisi, and Maasoumeh Jafarpour
Department of Chemistry, Faculty of Science, University of Birjand, Birjand, 97179-414 Iran

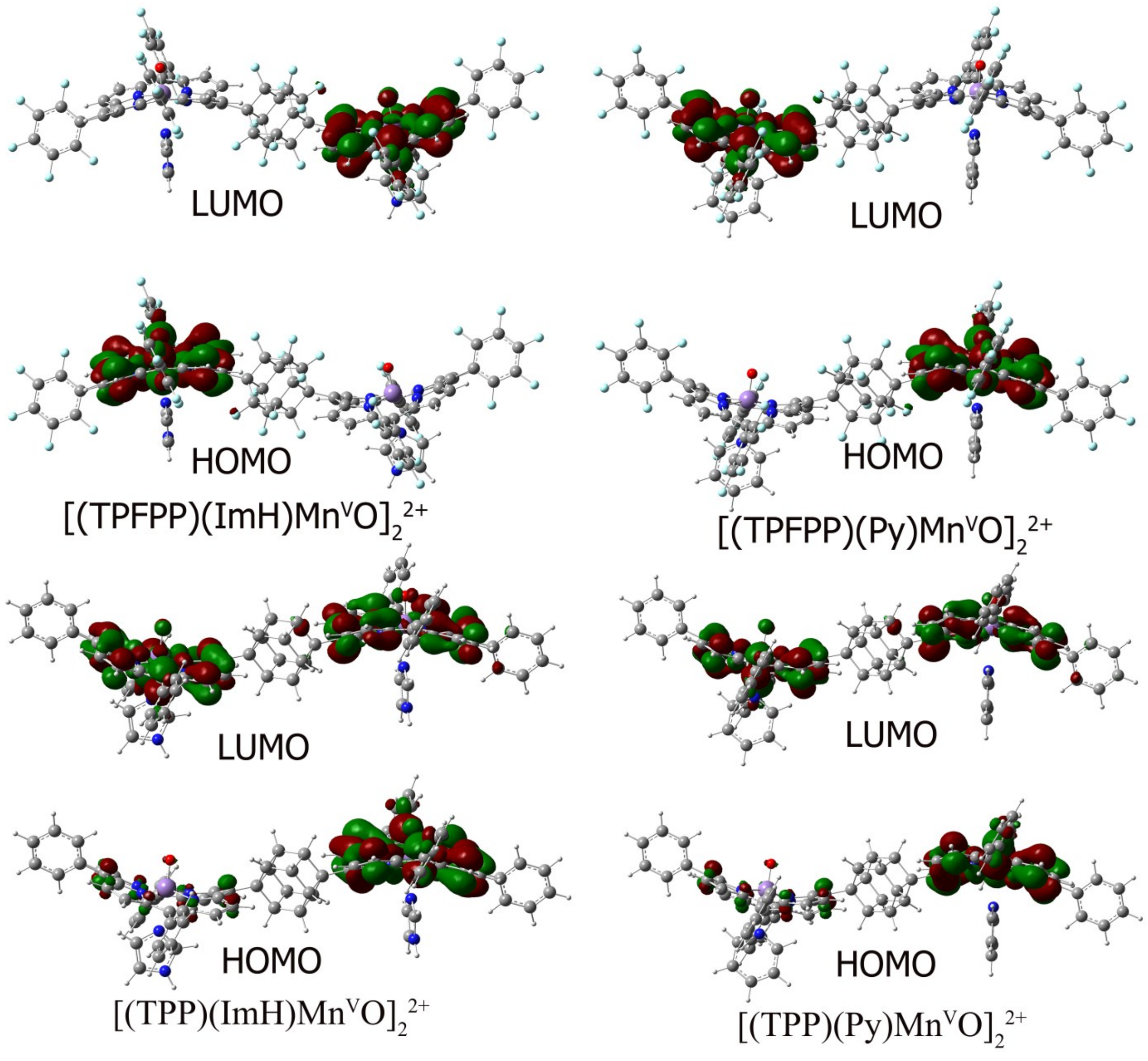

Figure S1. HOMO and LUMO compositions of the frontier molecular orbitals for $[(TPP)(ImH)Mn^{V}O]_2^{2+}$, $[(TPP)(Py)Mn^{V}O]_2^{2+}$, $[(TPFPP)(ImH)Mn^{V}O]_2^{2+}$ and $[(TPFPP)(Py)Mn^{V}O]_2^{2+}$ complexes.

Table S1. The calculated dipole moments (μ°) (in Debye), the highest occupied molecular orbital (HOMO) and lowest unoccupied molecular orbital (LUMO) energies, $\Delta E_{(H-L)}$, chemical hardness (η), chemical potential (μ) in terms of eV for $[(TPP)(N\text{-donor})MnO]_2^{2+}$ and $[(TPFPP)(N\text{-donor})MnO]_2^{2+}$ intermediates calculated in the gas phase.

Porphyrin	N-donor	μ°	E_{HOMO}	E_{LUMO}	$\Delta E_{(H-L)}$	η	μ
TPP	ImH	10.48	-8.74	-7.52	1.22	0.61	-8.13
	Py	6.55	-8.87	-7.58	1.28	0.64	-8.22
	Piperidine	2.75	-8.88	-7.66	1.21	0.60	-8.27
	none	0.79	-9.29	-7.66	1.62	0.81	-8.48
TPFPP	ImH	11.77	-9.71	-8.27	1.44	0.72	-8.99
	Py	8.62	-9.79	-8.31	1.48	0.74	-9.05
	Piperidine	4.20	-9.80	-8.33	1.47	0.73	-9.06
	none	0.31	-10.15	-8.43	1.72	0.86	-9.29

Table S2. The selected topological parameters of investigated π-complexes and the density of the total energy of electrons (H_C) and its two components, the kinetic(G_C) and potential (V_C) electron energy densities (in a.u.) in the gas phase.

Porphyrin	N-donor	Bond	ρ_{BCP}	$\nabla^2_{\rho BCP}$	G_C	V_C	H_C
TPP	ImH	Mn-O	0.3235	1.1627	0.6316	-0.9725	-0.3409
		$Mn\text{-}N_{(ax)}$	0.0396	0.1341	0.0355	-0.0376	-0.0020
	Py	Mn-O	0.3256	1.1443	0.6333	-0.9804	-0.3472
		$Mn\text{-}N_{(ax)}$	0.0270	0.0780	0.0200	-0.0205	-0.0005
	Piperidine	Mn-O	0.3236	1.1649	0.6321	-0.9729	-0.3409
		$Mn\text{-}N_{(ax)}$	0.0304	0.0768	0.0213	-0.0234	-0.0021
	none	Mn-O	0.3220	1.1385	0.6240	-0.9634	-0.3394
		$Mn\text{-}N_{(ax)}$	----	----	----	----	----
TPFPP	ImH	Mn-O	0.3265	1.1902	0.6430	-0.9884	-0.3454
		$Mn\text{-}N_{(ax)}$	0.0453	0.1514	0.0418	-0.0457	-0.0039
	Py	Mn-O	0.3275	1.1703	0.6418	-0.9910	-0.3492
		$Mn\text{-}N_{(ax)}$	0.0352	0.1053	0.0284	-0.0305	-0.0021
	Piperidine	Mn-O	0.3251	1.1790	0.6379	-0.9810	-0.3431
		$Mn\text{-}N_{(ax)}$	0.0334	0.0823	0.0237	-0.0268	-0.0031
	none	Mn-O	0.3276	1.1319	0.6357	-0.9884	-0.3527
		$Mn\text{-}N_{(ax)}$	----	----	----	----	----

Table S3. NPA charges, the second-order perturbation energy ($kcal.mol^{-1}$) and natural atomic orbital occupancies of Mn orbitals for $[(TPP)(N\text{-donor})MnO]_2^{2+}$ and $[(TPFPP)(N\text{-donor})MnO]_2^{2+}$ intermediates calculated in the gas phase.

Porphyrin	N-donor	q_{Mn}	$q_{N(ax)}$	q_O	$lpN_{(ax)} \rightarrow \sigma^*_{(Mn\text{-}O)}$	d_{xy}	d_{xz}	d_{yz}	$d_{x^2-y^2}$	d_{z^2}
TPP	ImH	1.359	-0.456	-0.298	6.71	1.014	0.946	0.910	1.940	1.043
	Py	1.363	-0.438	-0.292	5.43	1.006	0.948	0.909	1.931	1.038
	Piperidine	1.326	-0.246	-0.287	6.41	0.978	1.088	1.120	1.675	0.967
	None	1.245	----	-0.305	----	0.953	1.124	1.115	1.647	0.967
TPFPP	ImH	1.351	-0.749	-0.280	16.15	1.022	0.954	0.919	1.923	1.045
	Py	1.346	-0.449	-0.275	9.74	1.014	0.965	0.918	1.914	1.037
	Piperidine	1.320	-0.250	-0.272	10.47	0.983	1.132	1.144	1.625	0.959
	None	1.247	----	-0.277	----	0.957	1.166	1.127	1.599	0.956
